# Favorable short-term oncologic outcomes following laparoscopic surgery for small T4 colon cancer: a multicenter comparative study

**DOI:** 10.1186/s12957-020-02074-5

**Published:** 2020-11-13

**Authors:** Sung Sil Park, Joon Sang Lee, Hyoung-Chul Park, Sung Chan Park, Dae Kyung Sohn, Jae Hwan Oh, Kyung Su Han, Dong-Won Lee, Dong-Eun Lee, Sung-Bum Kang, Kyu Joo Park, Seung-Yong Jeong, on behalf of the Seoul Colorectal Research Group (SECOG)

**Affiliations:** 1 Center for Colorectal Cancer, National Cancer Center, Goyang, Korea; 2 Biostatistics Collaboration Team, Research Institute and Hospital, National Cancer Center, Goyang, Korea; 3 Department of Surgery, Seoul National University Bundang Hospital, Seongnam, Korea; 4 Department of Surgery, Seoul National University College of Medicine, Seoul, Korea

**Keywords:** Colon cancer, Laparoscopy, Open surgery, T4 cancer, Tumor size

## Abstract

**Background:**

Laparoscopic surgery for T4 colon cancer may be safe in selected patients. We hypothesized that small tumor size might preoperatively predict a good laparoscopic surgery outcome. Herein, we compared the clinicopathologic and oncologic outcomes of laparoscopic and open surgery in small T4 colon cancer.

**Methods:**

In a retrospective multicenter study, we reviewed the data of 449 patients, including 117 patients with tumors ≤ 4.0 cm who underwent surgery for T4 colon cancer between January 2014 and December 2017. We compared the clinicopathologic and 3-year oncologic outcomes between the laparoscopic and open groups. Survival curves were estimated using the Kaplan–Meier method and compared using the log-rank test. Univariate and multivariate analyses were performed using the Cox proportional hazards model. A *p* < 0.05 was considered statistically significant.

**Results:**

Blood loss, length of hospital stay, and postoperative morbidity were lower in the laparoscopic group than in the open group (median [range], 50 [0–700] vs*.* 100 [0–4000] mL, *p* < 0.001; 8 vs*.* 10 days, *p* < 0.001; and 18.0 vs*.* 29.5%, *p* = 0.005, respectively). There were no intergroup differences in 3-year overall survival or disease-free survival (86.6 vs*.* 83.2%, *p* = 0.180, and 71.7 vs*.* 75.1%, *p* = 0.720, respectively). Among patients with tumor size ≤ 4.0 cm, blood loss was significantly lower in the laparoscopic group than in the open group (median [range], 50 [0–530] vs*.* 50 [0–1000] mL, *p* = 0.003). Despite no statistical difference observed in the 3-year overall survival rate (83.3 vs*.* 78.7%, *p* = 0.538), the laparoscopic group had a significantly higher 3-year disease-free survival rate (79.2 vs*.* 53.2%, *p* = 0.012).

**Conclusions:**

Laparoscopic surgery showed similar outcomes to open surgery in T4 colon cancer patients and may have favorable short-term oncologic outcomes in patients with tumors ≤ 4.0 cm.

**Supplementary Information:**

The online version contains supplementary material available at 10.1186/s12957-020-02074-5.

## Background

Approximately 10–20% of patients with colon cancer are diagnosed with T4 colon cancer [1–3]. R0 resection is essential for curative surgery in T4 colon cancer, although R0 resection is not easily achieved in case of tumor invasion into the adjacent organs or structures. Several meta-analyses and randomized controlled trials [4–7] have reported that laparoscopic surgery is non-inferior to open surgery for colon cancer. However, in T4 colon cancer, the feasibility of laparoscopic surgery with regard to oncologic outcomes remains debatable. In addition, treatment guidelines recommend an open approach for pathological T4 colon cancer.

Several recent studies [8–10] have reported that laparoscopic surgery for T4 colon cancer had better short-term outcomes (e.g., less intraoperative blood loss and shorter hospital stay) than open surgery, as well as non-inferiority in oncologic outcomes. However, the exact clinical conditions wherein laparoscopic surgery for T4 colon cancer is feasible or harmful, with regard to oncologic outcomes, need to be ascertained. Studies [11, 12] have reported that a technical difficulty during laparoscopic surgery could threaten oncological safety, while tumor size is a factor that is known to influence the technical difficulty associated with tumor resection.

In T4 colon cancer, a laparoscopic approach seems to be superior in regard to clinical outcomes in cases where the tumor is easy to access or handle, such as with a small invasive tumor. However, large-sized tumors are more difficult to resect laparoscopically, which may increase the risk of tumor spillage. However, there is scant evidence of the comparative outcomes of laparoscopic and open surgery with respect to the tumor size in T4 colon cancer.

In this study, we investigated the hypothesis that tumor size may influence the preoperative prediction of a favorable outcome following a laparoscopic approach and evaluated the clinicopathologic and oncologic outcomes of laparoscopic and open surgery in patients with small T4 colon cancer.

## Methods

### Patient characteristics

A retrospective chart review and analysis of multicenter data were undertaken, including data from patients diagnosed with pathological T4 colon cancer who underwent curative surgery at three institutions between January 2014 and December 2017. Rectal cancer was defined as cancer in which the lower margin of the tumor was located within 15.0 cm above the anal verge, and patients with rectal cancer were excluded from this study. Moreover, patients with T1–3 colon cancer, a histological diagnosis indicating cancer other than adenocarcinoma, palliative surgery, inflammatory bowel disease, or hereditary colon cancer were excluded.

The patient characteristics and perioperative outcomes were analyzed, including age, gender, body mass index (BMI), American Society of Anesthesiologists (ASA) score, preoperative carcinoembryonic antigen level, tumor location, operative time, blood loss, intraoperative transfusion, length of hospital stay, and postoperative morbidity. The pathologic features that were analyzed included tumor size, T stage, nodal status, angiolymphatic invasion, venous invasion, perineural invasion, adjacent organ resection, and R0 resection. The tumor size was measured on the basis of the long diameter of the tumor in the pathologic specimen. Patients with ASA scores of 1–2 and 3–4 were included in the same group for analysis. The tumor location was divided into the right (from the cecum to the transverse colon) and left (from the splenic flexure to the sigmoid colon) sides. The nodal status was classified as the absence (N0) or presence (N+) of metastatic regional lymph node(s).

All surgeons who participated in the study were experts who had performed laparoscopic or open colorectal surgery for > 10 years. Laparoscopic or open surgery was performed according to each surgeon’s preference.

### Outcomes

The primary outcome of this study was the comparison of oncologic outcomes, including 3-year overall survival (OS) and 3-year disease-free survival (DFS), between the laparoscopic and open groups. With regard to DFS, we additionally analyzed locoregional recurrence-free survival (LRFS) and distant recurrence-free survival (DRFS) in the entire cohort and in patients with tumor size ≤ 4.0 cm. The secondary outcome was the R0 resection rate. Small T4 colon cancer was defined as tumor size ≤ 4.0 cm, which may be advantageous in laparoscopic surgery with small incisions.

OS was defined as the time from surgery to death, and DFS was defined as the time from surgery to any recurrence, secondary cancer, or death. R0 resection was defined as a microscopically margin-negative resection in which no gross or microscopic tumor remains in the primary tumor bed. A negative margin was defined as a margin of normal tissue > 1.0 mm from the edge of the tumor.

### Statistical analyses

Data are reported as mean ± standard deviation or median (range) for continuous variables and as number (percentage) for categorical variables. The comparison of the variables between the laparoscopic and open groups was performed using the independent *t* test or Wilcoxon rank sum test and chi-square test or Fisher’s exact test. Survival curves were analyzed using the Kaplan–Meier method, and the intergroup differences were compared using the log-rank test. The univariate Cox proportional hazards model was used to determine prognostic factors for OS and DFS. Variables with *p* < 0.05 in the univariate analysis were included in the multivariate analysis. The backward elimination method, with *p* > 0.05 as the criterion for removal, was performed for the multivariate analysis. After significant clinical variables were adjusted, the prognosis of the surgical procedure was evaluated. A *p* value < 0.05 was considered statistically significant. Statistical analyses were performed using R (version 3.6.2; The R Foundation for Statistical Computing, Vienna, Austria) and SAS (version 9.4; SAS Institute Inc., Cary, NC, USA).

## Results

### Patient characteristics

**Fig. 1:**
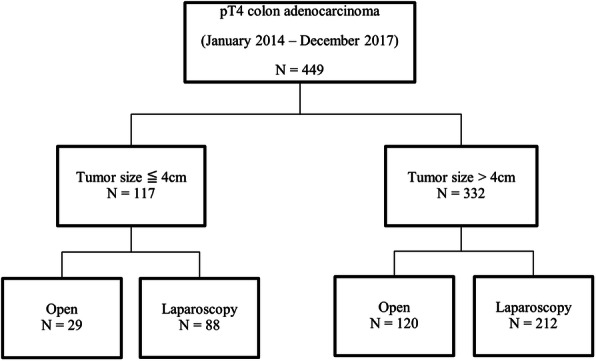
Flowchart of patient enrollment

A total of 449 patients were included and classified according to tumor size; 117 and 332 patients had tumors of ≤ 4.0 and > 4.0 cm, respectively. In the ≤ 4.0-cm group, 88 and 29 patients underwent laparoscopic and open surgery, respectively. In the > 4.0-cm group, 194 and 138 patients underwent laparoscopic and open surgery, respectively (Fig. 1). Twenty-one patients who converted from laparoscopic to open surgery were included in the open group.

Patients in the laparoscopic group had a higher BMI (23.7 vs*.* 22.0 kg/m^2^, *p* < 0.001) and a lower proportion of patients in this group had an ASA score of > 2 (4 vs*.* 14.1%, *p* < 0.001) than in the open group. The proportions of blood loss and postoperative transfusion were lower in the laparoscopic group than in the open group (50 vs*.* 100 mL, *p* < 0.001, and 0.7 vs*.* 12.8%, *p* < 0.001, respectively). Patients in the laparoscopic group had a shorter hospital stay (8 vs*.* 10 days, *p* < 0.001) and a lower postoperative morbidity (18 vs*.* 29.5%, *p* = 0.005) than those in the open group (Table 1).

**Table 1:** Patient characteristics and perioperative outcomes

Variable		Open (*N* = 149)	Laparoscopy (*N* = 300)	*p* value
Age (years)		64.9 ± 12.8	63.6 ± 12.6	0.298^a^
Gender	Male	82 (55.0)	172 (57.3)	0.643^c^
	Female	67 (45.0)	128 (42.7)	
BMI (kg/m^2^)		22.0 ± 3.3	23.7 ± 3.5	< .001^a^
ASA score	1, 2	128 (85.9)	288 (96)	< .001^c^
	3, 4	21 (14.1)	12 (4)	
Preoperative CEA (ng/ml)		3.3 (0.5–338)	4.3 (0.4–543)	0.126^b^
Location	Right	64 (43.0)	142 (47.3)	0.380^c^
	Left	85 (57.0)	158 (52.7)	
Operative time (min)		141 (43–520)	160 (50–460)	0.007^b^
Blood loss (ml)		100 (0–4000)	50 (0–700)	< .001^b^
Transfusion	No	130 (87.3)	298 (99.3)	< .001^d^
	Yes	19 (12.8)	2 (0.7)	
Hospital stay (days)		10 (5–45)	8 (4–158)	< .001^b^
Postoperative morbidity	No	105 (70.5)	246 (82.0)	0.005^c^
	Yes	44 (29.5)	54 (18.0)	
Postoperative morbidity type	Ileus	7 (15.9)	15 (27.8)	
	Urinary retention	2 (4.6)	5 (9.3)	
	Anastomotic leakage	0 (0)	3 (5.6)	
	Surgical site infection	17 (38.6)	15 (27.8)	
	Pneumonia	5 (11.4)	2 (3.7)	
	Sepsis	3 (6.8)	3 (5.6)	
	Others	10 (22.7)	11 (20.4)	
**Clavien–Dindo classification**	1, 2	34 (77.3)	42 (77.8)	0.953^c^
	3	10 (22.7)	12 (22.2)	
Adjuvant chemotherapy (*N* = 329)	No	20 (28.6)	39 (15.1)	0.009 ^c^
	Yes	50 (71.4)	220 (78.7)	

^a^Two-sample *t* test

^b^Wilcoxon rank sum test

^c^Chi-square test

^d^Fisher’s exact test

Data are expressed as mean ± standard deviation or median (range) for continuous variables and as number (percentage) for categorical variables

*ASA* American Society of Anesthesiologists, *BMI* body mass index, *CEA* carcinoembryonic antigen

### Pathologic and oncologic outcomes

Patients in the laparoscopic group had smaller tumors (5.2 vs*.* 6 cm, *p* < 0.001) and a lower T4b rate (17.3 vs*.* 43.0%, *p* < 0.001) than those in the open group. Angiolymphatic, venous, and perineural invasion were more common in the laparoscopic group than in the open group (74 vs*.* 43.0%, *p* < 0.001; 50 vs*.* 34.9%, *p* = 0.003; and 78 vs*.* 62.4%, *p* = 0.001, respectively). Similarly, the adjacent organ resection rate was lower in the laparoscopic group than in the open group (6 vs*.* 28.2%, *p* < 0.001).

**Fig. 2:**
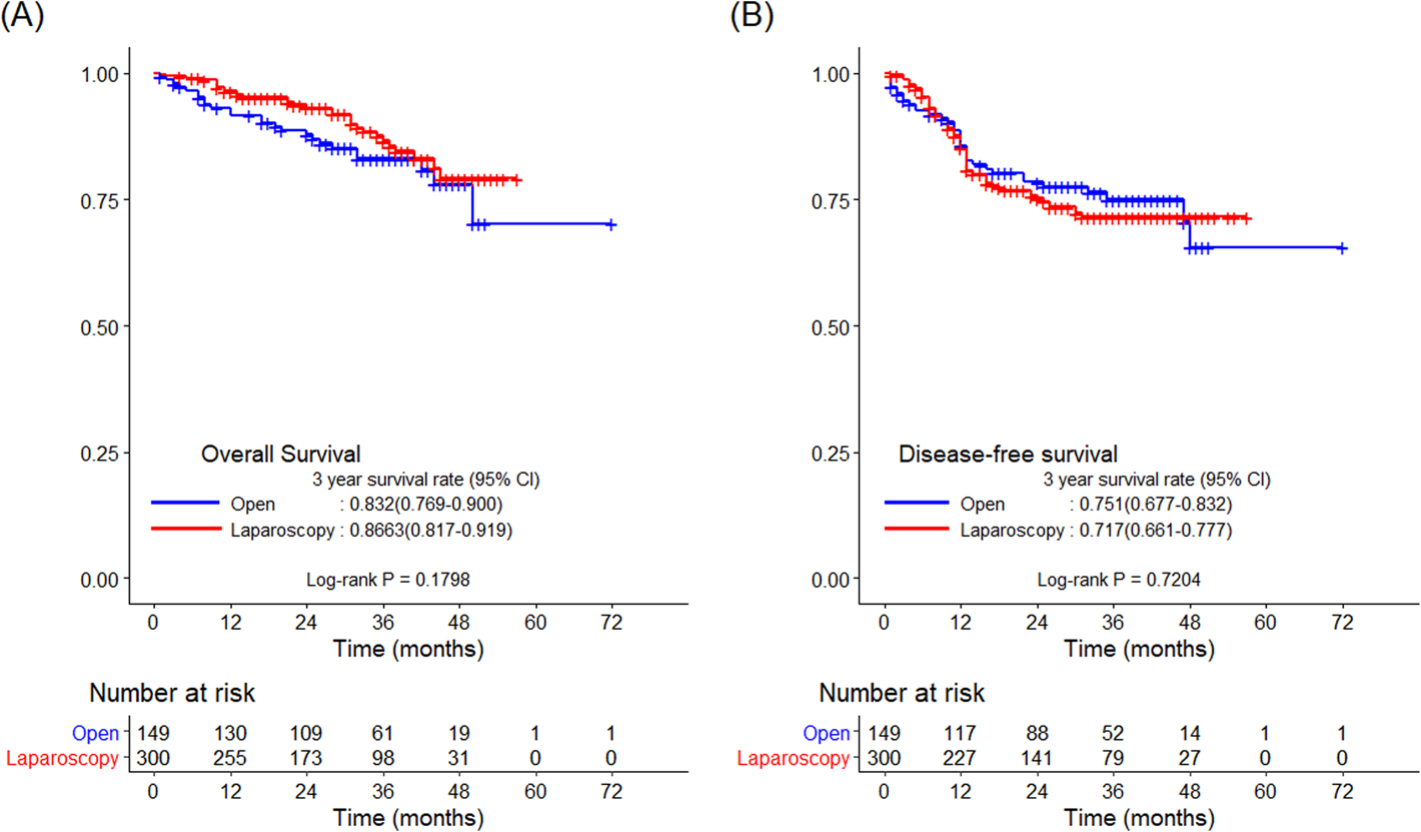
Kaplan–Meier curves comparing survival outcomes between the laparoscopic and open groups. **a** 3-year OS and **b** 3-year DFS. DFS, disease-free survival; OS, overall survival

**Fig. 3:**
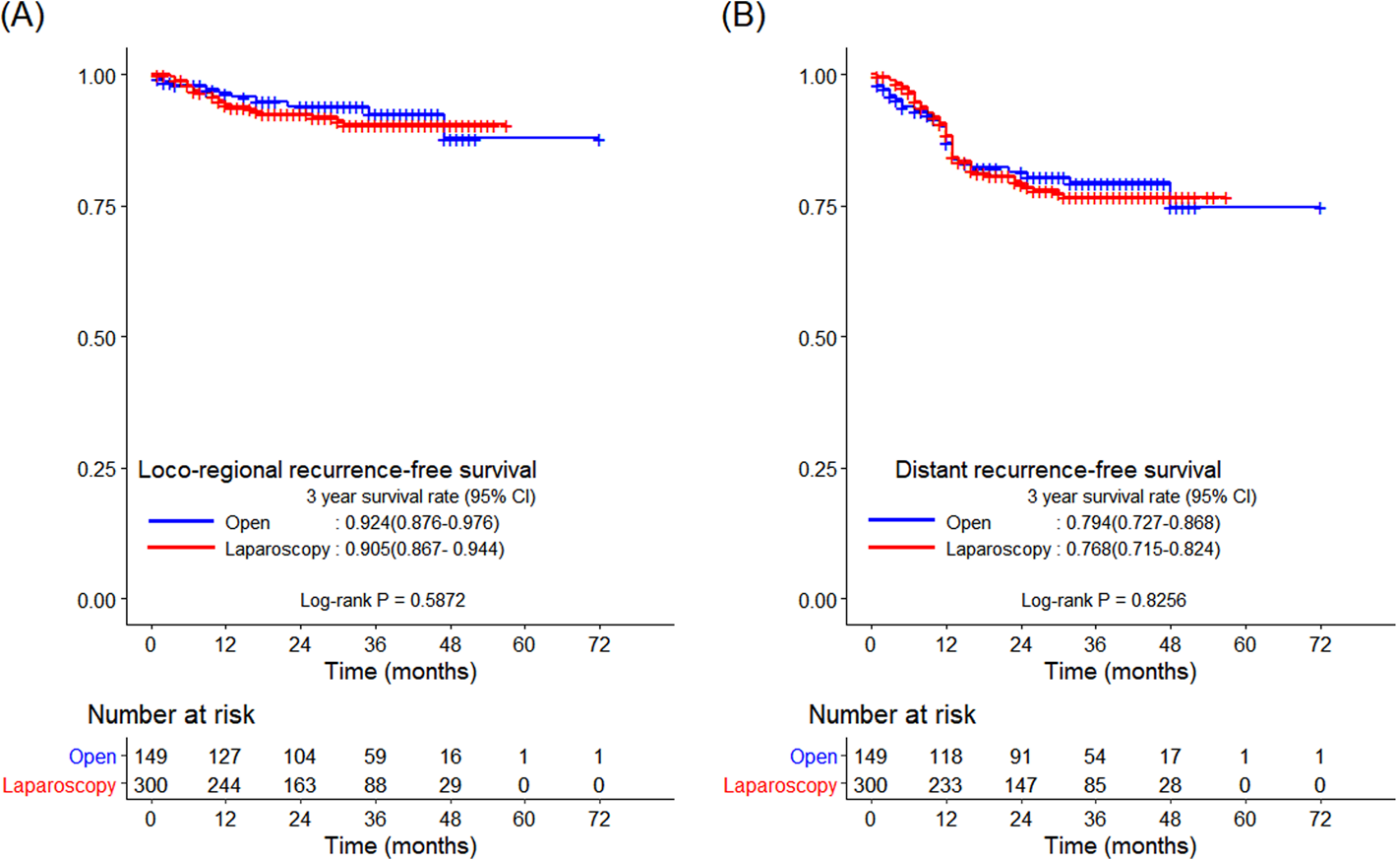
Kaplan–Meier curves comparing survival outcomes between the laparoscopic and open groups. **a** 3-year LRFS and **b** 3-year DRFS. DRFS, distant recurrence-free survival; LRFS, locoregional recurrence-free survival

The R0 resection rate did not differ significantly between the two groups (94.0 vs*.* 97.3%, *p* = 0.078; Table 2). The median follow-up period was 34 months. There were no significant intergroup differences with regard to the 3-year OS and DFS rates (83.2 vs*.* 86.6%, *p* = 0.180 and 75.1 vs*.* 71.7%, *p* = 0.720, respectively; Fig. 2). The 3-year LRFS and DRFS rates also did not differ significantly between the two groups (92.4 vs*.* 90.5%, *p* = 0.587 and 79.4 vs*.* 76.8%, *p* = 0.826, respectively; Fig. 3).

**Table 2:** Pathologic features and oncologic outcomes

Variable		Open (*N* = 149)	Laparoscopy (*N* = 300)	*p* value
Tumor size (cm)		6 (2–30)	5.2 (0.9–14.5)	< .001^a^
Node state	N0	53 (35.6)	85 (28.3)	0.118^b^
	N+	96 (64.4)	215 (71.7)	
T stage	T4a	85 (57.0)	248 (82.7)	< .001^b^
	T4b	64 (43.0)	52 (17.3)	
Angiolymphatic invasion	Not identified	85 (57.0)	78 (26.0)	< .001^b^
	Present	64 (43.0)	222 (74.0)	
Venous invasion	Not identified	97 (65.1)	150 (50.0)	0.003^b^
	Present	52 (34.9)	150 (50.0)	
Perineural invasion	Not identified	56 (37.6)	66 (22.0)	0.001^b^
	Present	93 (62.4)	234 (78.0)	
Combined resection	No	107 (71.8)	282 (94.0)	< .001^b^
	Yes	42 (28.2)	18 (6.0)	
R0 resection rate		140 (94)	292 (97.3)	0.078^b^
Harvested lymph nodes	*N* = 298	29 (5–117)	29 (7–244)	0.677^a^
Proximal margin	*N* = 447	14 (0.5–174)	10.7 (1.5–119.8)	0.004^a^
Distal margin		8 (0–125.5)	8.3 (0.4–101)	0.532^a^
Radial margin	*N* = 100	0.4 (0–8.5)	0.4 (0–4)	0.895^a^

^a^Wilcoxon rank sum test

^b^Chi-square test

Data are expressed as median (range) for continuous variables and as number (percentage) for categorical variables

### Outcomes of small T4 colon cancer

Table 3 shows the clinical characteristics and perioperative outcomes of patients with tumor size ≤ 4.0 cm. The laparoscopic group had a higher BMI (23.9 vs*.* 22.3 kg/m^2^, *p* = 0.026) and less blood loss (50 [0–1000] vs*.* 50 [0–530] mL, *p* = 0.03) than the open group. Other variables did not differ significantly between the two groups.

**Table 3:** Patient characteristics and perioperative outcomes in patients with tumor size ≤ 4.0 cm

Variable		Open (*N* = 29)	Laparoscopy (*N* = 88)	*p* value
Age (years)		62.2 ± 12.0	65.1 ± 12.8	0.287^a^
Gender	Male	14 (48.3)	46 (52.3)	0.709^c^
	Female	15 (51.7)	42 (47.7)	
BMI (kg/m^2^)		22.3 ± 3.2	23.9 ± 3.2	0.026^a^
ASA score	1, 2	28 (96.6)	87 (98.9)	0.436^d^
	3, 4	1 (3.5)	1 (1.1)	
Preoperative CEA (ng/ml)		2.4 (0.6–54.9)	3.3 (0.4–138)	0.270^b^
Location	Right	12 (41.4)	40 (45.5)	0.702^c^
	Left	17 (58.6)	48 (54.6)	
Operative time (min)		75 (45–505)	148 (85–460)	< .001^b^
Blood loss (ml)		50 (0–1000)	50 (0–530)	0.003^b^
Transfusion	No	27 (93.1)	88 (100)	0.060^d^
	Yes	2 (6.9)	0 (0)	
Hospital stay (days)		8 (5–36)	8 (4–31)	0.942^b^
Postoperative morbidity	No	23 (79.3)	70 (79.6)	0.978^c^
	Yes	6 (20.7)	18 (20.5)	
Postoperative morbidity type	Ileus	1 (16.7)	6 (33.3)	
	Urinary retention	0 (0)	2 (11.1)	
	Anastomotic leakage	0 (0)	1 (5.6)	
	Surgical site infection	3 (50)	4 (22.2)	
	Sepsis	0 (0)	1 (5.6)	
	Others	2 (33.3)	4 (22.2)	
Clavien–Dindo classification	1, 2	6 (100)	14 (77.8)	0.539^d^
	3	0 (0)	4 (22.2)	
Adjuvant chemotherapy	No	1 (20.0)	18 (22.9)	1.000^c^
(*N* = 77)	Yes	4 (80.0)	54 (77.1)	

^a^Two-sample *t* test

^b^Wilcoxon rank sum test

^c^Chi-square test

^d^Fisher’s exact test

Data are expressed as mean ± standard deviation or median (range) for continuous variables and as number (percentage) for categorical variables

*ASA* American Society of Anesthesiologists, *BMI* body mass index, *CEA* carcinoembryonic antigen

Table 4 presents the pathologic features and oncologic outcomes of patients with tumor size ≤ 4.0 cm. Patients in the laparoscopic group were more likely to have angiolymphatic invasion than those in the open group (77.3 vs*.* 37.9%, *p* < 0.001).

**Table 4:** Pathologic features and oncologic outcomes in patients with tumor size ≤ 4.0 cm

Variable		Open (*N* = 29)	Laparoscopy (*N* = 88)	*p* value
Tumor size (cm)		3.5 (2–4)	3.4 (0.9–4)	0.208^a^
Node state	N0	7 (24.1)	22 (25.0)	0.926^b^
	N+	22 (75.9)	66 (75.0)	
T stage	T4a	26 (89.7)	85 (96.6)	0.161^c^
	T4b	3 (10.3)	3 (3.4)	
Angiolymphatic invasion	Not identified	18 (62.1)	20 (22.7)	< .001^b^
	Present	11 (37.9)	68 (77.3)	
Venous invasion	Not identified	19 (65.5)	50 (56.8)	0.409^b^
	Present	10 (34.5)	38 (43.2)	
Perineural invasion	Not identified	7 (24.1)	11 (12.5)	0.146^c^
	Present	22 (75.9)	77 (87.5)	
Combined resection	No	27 (93.1)	86 (97.7)	0.256^c^
	Yes	2 (6.9)	2 (2.3)	
R0 resection rate		29 (100)	88 (100)	-
Harvested lymph nodes	*N* = 83	18 (8–60)	25 (7–107)	0.057^a^
Proximal margin	*N* = 116	8.5 (2–43)	10.1 (1.5–48)	0.198^a^
Distal margin		5.5 (0–27.5)	7.5 (0.4–50)	0.241^a^
Radial margin	*N* = 10	1.4 (0.4–2.3)	1.1 (0.1–4)	0.896^a^

^a^Wilcoxon rank sum test

^b^Chi-square test

^c^Fisher’s exact test

Data are expressed as median (range) for continuous variables and as number (percentage) for categorical variables

**Fig. 4:**
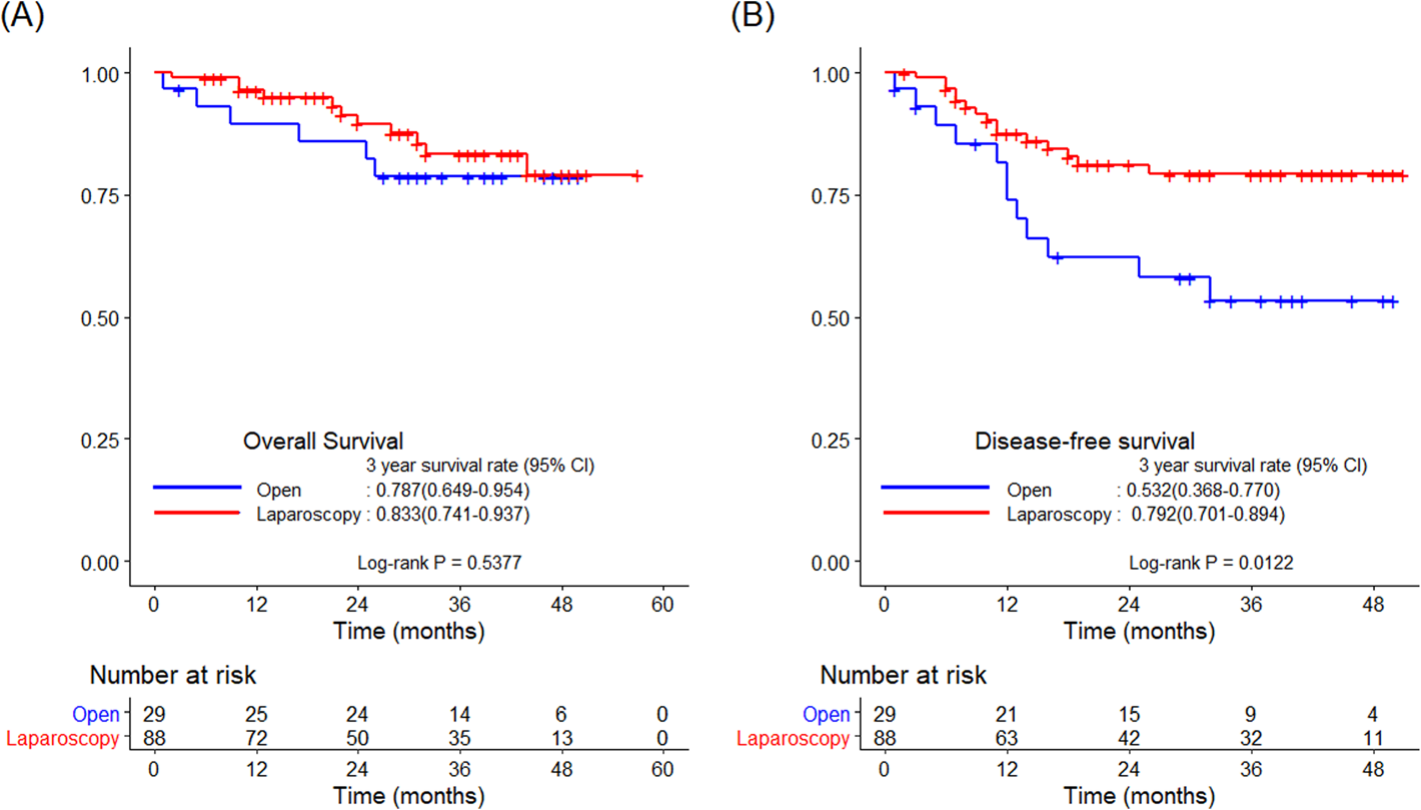
Kaplan–Meier curves comparing survival outcomes between the laparoscopic and open groups. **a** 3-year OS and **b** 3-year DFS in patients with tumor size ≤ 4.0 cm. DFS, disease-free survival; OS, overall survival

**Fig. 5:**
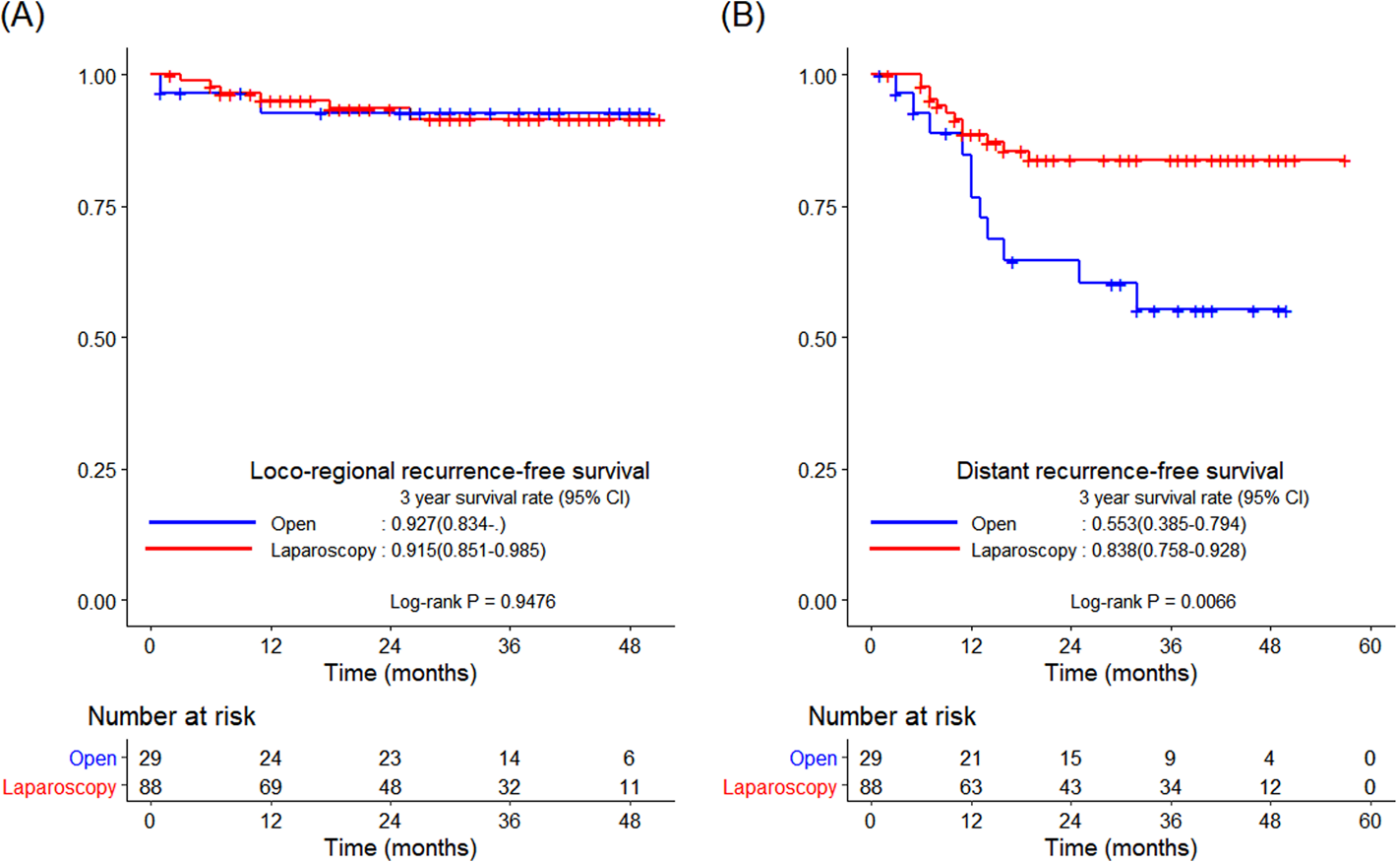
Kaplan–Meier curves comparing survival outcomes between the laparoscopic and open groups. **a** 3-year LRFS and **b** 3-year DRFS in patients with tumor size ≤ 4.0 cm. DRFS, distant recurrence-free survival; LRFS, locoregional recurrence-free survival

R0 resection was performed in all patients in both groups. In patients with tumor size ≤ 4.0 cm, the 3-year OS rate did not differ significantly between the two groups (78.7 vs*.* 83.3%, *p* = 0.538). However, the 3-year DFS rate was higher in the laparoscopic group than in the open group (79.2 vs*.* 53.2%, *p* = 0.012; Fig. 4). The 3-year LRFS rate did not differ significantly between the two groups (92.7 vs*.* 91.5%, *p* = 0.948). In contrast, the DRFS rate was higher in the laparoscopic group than in the open group (83.8 vs*.* 55.3%, *p* = 0.007; Fig. 5).

## Discussion

Although the safety of laparoscopic surgery for colon cancer had been demonstrated in several studies [4–7], the safety of this surgical approach is controversial in T4 colon cancer. Several studies have suggested that a laparoscopic approach in T4 colon cancer may be feasible in some patients. Few studies have provided useful indications for laparoscopic surgery in T4 colon cancer. Klaver et al*.* [2] reported that laparoscopic surgery for T4a tumors might be safe. However, the pathologic features would not be helpful in determining the indication of laparoscopic surgery preoperatively. Park et al*.* [13] found the laparoscopic approach to be feasible for left-sided T4 colon cancer. Nevertheless, a useful predictor is still necessary to preoperatively determine the safety of laparoscopic surgery for T4 cancer.

In this study, the clinicopathologic and oncologic outcomes of laparoscopic surgery for T4 colon cancer were generally comparable to those of open surgery. The laparoscopic approach, especially for small T4 tumors, had better 3-year DFS rates than open surgery. To adjust for confounding variables, we analyzed the Cox proportional hazards regression model for OS and DFS in the entire cohort and in patients with tumor size ≤ 4.0 cm. Laparoscopic surgery had better DFS rates in patients with tumor size ≤ 4.0 cm (*p* = 0.020) (Additional files 1 and 2).

A previous study [14] has reported that malignant cells are intraoperatively exfoliated from the tumor during resection and spread to the peritoneal surface and portal vein system. This can be prevented by minimizing tumor manipulation, e.g., through laparoscopic surgery. Lacy et al*.* [15] showed better cancer-related survival with laparoscopic colectomy than open surgery for non-metastatic colon cancer in a randomized clinical trial, as did our study. When laparoscopic surgery is conducted by an experienced surgeon, tumor spillage, and spread may be prevented in some patients.

As tumor size increases, some technical challenges arise with regard to laparoscopic surgery, because it reduces the working space, narrows the operative visual field, increases bleeding, and makes the tumor difficult to remove. Moreover, larger tumors increase the risk of tumor spillage, thereby increasing peritoneal seeding or trocar-site recurrence. Our data show that the 3-year OS and DFS rates in patients with tumor size > 4.0 cm are not significantly different between the two groups (84.4 vs*.* 87.8%, *p* = 0.22 and 80.6 vs*.* 68.7%, *p* = 0.091, respectively), suggesting that the laparoscopic approach is more feasible in patients with small tumors than in those with larger tumors.

Laparoscopic surgery is better than open surgery in regard to perioperative outcomes. In previous studies [1, 16, 17] comparing laparoscopic and open surgery in T4 colon cancer, laparoscopic surgery was associated with less intraoperative blood loss, which has been proven to be a predictor of long-term survival [18, 19]. Some studies [20, 21] have shown that hospital stays are shorter in patients who undergo laparoscopic surgery. In this study, patients in the laparoscopic group had less intraoperative blood loss and shorter hospital stays than those in the open group.

In a previous study [3] of T4 colon cancer, the conversion rate from laparoscopic to open surgery was reported to be in the range of 7.1–28.2%. Converted patients have high postoperative morbidity and adverse effects on long-term oncologic outcomes [22]. In the present study, the overall conversion rate was 7%, and the conversion rate for patients with tumor size ≤ 4.0 cm was 2.3%. The low conversion rate might be responsible for the better oncologic outcomes of laparoscopic surgery.

In this study, the 3-year DFS rate of patients in the open group with tumor size ≤ 4.0 cm was 53.2%, which was much lower than the 75.1% for all patients in the open group. This result is similar to that of the study by Huang et al*.* [23], which reported that a smaller tumor size was associated with a decreased survival in the T4b subset of colon cancer patients. Huang et al*.* [23] suggested that small tumors in T4b patients may reflect a more biologically aggressive phenotype. Another plausible explanation is that surgeons may have conducted more aggressive surgery for larger tumors. In the present study, the rate of multi-visceral resection was 28.2% in the entire open group, but only 6.9% in the small tumor group. Although R0 resection was accomplished in all patients with small tumors, it is possible that disseminated lesions remained in adjacent organs. These may have contributed to the worse 3-year DFS rate in patients with tumor size ≤ 4.0 cm in the open group.

The limitations of this study are as follows. As this was a retrospective study, the choice of surgical approach may have been influenced by the patient’s condition or tumor progression. First, this study was conducted on the basis of the pathological T4 instead of the clinical T4, although the former cannot be used to determine the surgical approach preoperatively. Engelmann et al*.* [24] reported that the computed tomography accuracy of T4 staging in colon cancer was only 70–77%, although further studies are needed in patients with clinical T4 colon cancer. Second, more patients had higher ASA scores in the open group. This may have affected OS or DFS. However, in patients with tumor size ≤ 4.0 cm, there was no intergroup difference in ASA scores. Third, the T4b rate and number of adjacent organ resections were higher in the open group. Thus, it is apparent that open surgery was chosen for patients with more advanced tumors. However, there were no intergroup differences in the T4b rate and number of adjacent organ resections in patients with tumor size ≤ 4.0 cm.

## Conclusions

Although laparoscopic surgery showed similar outcomes in T4 colon cancer to open surgery, the former appears to have favorable short-term oncologic outcomes in patients with tumor size ≤ 4.0 cm. Prospective large-scale studies are needed to identify improved oncologic outcomes of laparoscopic surgery for small T4 colon cancer.

## Supplementary Information

**Additional file 1.** Univariate and multivariate analysis of OS and DFS.

**Additional file 2.** Univariate and multivariate analysis of OS and DFS in patients with tumor size ≤4.0 cm.

## Data Availability

The datasets used and/or analyzed during the current study are available from the corresponding author on reasonable request.

## Abbreviations

ASA: American Society of Anesthesiologists
BMI: Body mass index
DFS: Disease-free survival
DRFS: Distant recurrence-free survival
LRFS: Locoregional recurrence-free survival
OS: Overall survival
