# Supplementary material for: Favorable short-term oncologic outcomes following laparoscopic surgery for small T4 colon cancer: a multicenter comparative study
Source: World J Surg Oncol. 2020 Nov 13;18:299. doi: 10.1186/s12957-020-02074-5 (PMC7666454; doi:10.1186/s12957-020-02074-5)
Supplement: Supplementary file 1 — Additional file 1. Univariate and multivariate analysis of OS and DFS. [file 12957_2020_2074_MOESM1_ESM.docx]

**Additional file 1.** Univariate and multivariate analysis of OS and DFS

| Variable | | | OS | | | | | | | | | | | | | | | DFS | | | | | | | | | | | | | |
| --- | --- | --- | --- | --- | --- | --- | --- | --- | --- | --- | --- | --- | --- | --- | --- | --- | --- | --- | --- | --- | --- | --- | --- | --- | --- | --- | --- | --- | --- | --- | --- |
|  |  |  | N(EVENT) | | Univariate | | | | | Multivariate (p<0.05) | | | | Adjusted clinical variable | | | | N(EVENT) | | Univariate | | | | Multivariate (p<0.05) | | | | Adjusted clinical variable | | | |
|  |  |  |  |  | OR (95% CI) | | *p*-value | | OR (95% CI) | | | *p*-value | | OR (95% CI) | | *p*-value | |  | | OR (95% CI) | | *p*-value | | OR (95% CI) | | *p*-value | | OR (95% CI) | | *p*-value | |
| Surgery | Open | 149(25) | | 1(ref.) | | ─ | | ─ | | | ─ | | 1(ref.) | | ─ | | 149(34) | | 1(ref.) | | ─ | | ─ | | ─ | | 1(ref.) | | ─ | |  |
|  | Laparoscopic | 300(31) | | 0.699  (0.412─1.184) | | 0.183 | | ─ | | | ─ | | 0.802  (0.458─1.404) | | 0.4396 | | 300(70) | | 1.079  (0.716─1.626) | | 0.717 | | ─ | | ─ | | 0.915  (0.604─1.386) | | 0.6751 | |  |
| Age (years) |  | 449(56) | | 1.038  (1.014─1.063) | | 0.002^**^ | | 1.035  (1.010─1.062) | | | 0.0067^**^ | | 1.035  (1.009─1.061) | | 0.0077^**^ | | 449(104) | | 1.003  (0.987─1.018) | | 0.751 | | ─ | | ─ | | ─ | | ─ | |  |
| Gender | Male | 254(27) | | 1(ref.) | | ─ | | ─ | | | ─ | | ─ | | ─ | | 254(55) | | 1(ref.) | | ─ | | ─ | | ─ | | ─ | | ─ | |  |
|  | Female | 195(29) | | 1.551  (0.918─2.621) | | 0.101 | | ─ | | | ─ | | ─ | | ─ | | 195(49) | | 1.243  (0.846─1.828) | | 0.268 | | ─ | | ─ | | ─ | | ─ | |  |
| BMI (kg/m^2^) |  | 449(56) | | 0.945  (0.874─1.022) | | 0.160 | | ─ | | | ─ | | ─ | | ─ | | 449(104) | | 0.979  (0.926─1.036) | | 0.470 | | ─ | | ─ | | ─ | | ─ | |  |
| ASA score | 1–2 | 416(48) | | 1(ref.) | | ─ | | ─ | | | ─ | | ─ | | ─ | | 416(94) | | 1(ref.) | | ─ | | ─ | | ─ | | ─ | | ─ | |  |
|  | 3–4 | 33(8) | | 2.735  (1.291─5.794) | | 0.009^**^ | | ─ | | | ─ | | ─ | | ─ | | 33(10) | | 1.674  (0.872─3.216) | | 0.122 | | ─ | | ─ | | ─ | | ─ | |  |
| Preoperative CEA (10 ng/mL) | | | 430(52) | | 1.057  (1.020─1.097) | | 0.003^**^ | | 1.064  (1.024─1.105) | | | 0.0015^**^ | | 1.061  (1.021─1.103) | | 0.0027^**^ | | 430(102) | | 1.036  (1.002─1.072) | | 0.038^*^ | | ─ | | ─ | | ─ | | ─ | |
| Location | Right | 206(29) | | 1(ref.) | | ─ | | ─ | | | ─ | | ─ | | ─ | | 206(44) | | 1(ref.) | | ─ | | ─ | | ─ | | ─ | | ─ | |  |
|  | Left | 243(27) | | 0.714  (0.423─1.207) | | 0.209 | | ─ | | | ─ | | ─ | | ─ | | 243(60) | | 1.103  (0.747─1.628) | | 0.622 | | ─ | | ─ | | ─ | | ─ | |  |
| Operative time (min) | | | 449(56) | | 0.999  (0.996─1.002) | | 0.698 | | ─ | | | ─ | | ─ | | ─ | | 449(104) | | 1.000  (0.998─1.002) | | 0.966 | | ─ | | ─ | | ─ | | ─ | |
| Blood loss (10 mL) | | | 449(56) | | 0.996  (0.985─1.008) | | 0.521 | | ─ | | | ─ | | ─ | | ─ | | 449(104) | | 0.997  (0.989─1.005) | | 0.487 | | ─ | | ─ | | ─ | | ─ | |
| Transfusion | No | 428(52) | | 1(ref.) | | ─ | | ─ | | | ─ | | ─ | | ─ | | 449(104) | | 1(ref.) | | ─ | | ─ | | ─ | | ─ | | ─ | |  |
|  | Yes | 21(4) | | 1.189  (0.429─3.295) | | 0.739 | | ─ | | | ─ | | ─ | | ─ | | 428(99) | | 0.889  (0.362─2.185) | | 0.797 | | ─ | | ─ | | ─ | | ─ | |  |
| Hospital stay (days) | | | 449(56) | | 1.032  (1.020─1.044) | | <0.001^***^ | | 1.030  (1.017─1.043) | | | <0.0001^***^ | | 1.030  (1.017─1.043) | | <0.0001^***^ | | 21(5) | | 1.008  (0.986─1.031) | | 0.475 | | ─ | | ─ | | ─ | | ─ | |
| Postoperative morbidity | No | 351(44) | | 1(ref.) | | ─ | | ─ | | | ─ | | ─ | | ─ | | 351(82) | | 1(ref.) | | ─ | | ─ | | ─ | | ─ | | ─ | |  |
|  | Yes | 98(12) | | 0.962  (0.508─1.822) | | 0.905 | | ─ | | | ─ | | ─ | | ─ | | 98(22) | | 0.986  (0.615─1.579) | | 0.952 | | ─ | | ─ | | ─ | | ─ | |  |
| Conversion | No | 428(55) | | 1(ref.) | | ─ | | ─ | | | ─ | | ─ | | ─ | | 428(98) | | 1(ref.) | | ─ | | ─ | | ─ | | ─ | | ─ | |  |
|  | Yes | 21(1) | | 0.497  (0.069─3.600) | | 0.489 | | ─ | | | ─ | | ─ | | ─ | | 21(6) | | 1.542  (0.676─3.520) | | 0.304 | | ─ | | ─ | | ─ | | ─ | |  |
| Tumor size (cm) |  | 449(56) | | 0.937  (0.842─1.042) | | 0.232 | | ─ | | | ─ | | ─ | | ─ | | 449(104) | | 0.985  (0.918─1.056) | | 0.669 | | ─ | | ─ | | ─ | | ─ | |  |
| Nodal status | N0 | 138(11) | | 1(ref.) | | ─ | | ─ | | | ─ | | ─ | | ─ | | 138(18) | | 1(ref.) | | ─ | | 1(ref.) | | ─ | | 1(ref.) | | ─ | |  |
|  | N+ | 311(45) | | 1.852  (0.958─3.581) | | 0.067 | | ─ | | | ─ | | ─ | | ─ | | 311(86) | | 2.338  (1.406─3.886) | | 0.001^**^ | | 1.906  (1.130─3.216) | | 0.016^*^ | | 1.917  (1.135─3.236) | | 0.0149^*^ | |  |
| T stage | T4a | 333(42) | | 1(ref.) | | ─ | | ─ | | | ─ | | ─ | | ─ | | 333(79) | | 1(ref.) | | ─ | | ─ | | ─ | | ─ | | ─ | |  |
|  | T4b | 116(14) | | 1.029  (0.562─1.886) | | 0.926 | | ─ | | | ─ | | ─ | | ─ | | 116(25) | | 0.924  (0.589─1.449) | | 0.731 | | ─ | | ─ | | ─ | | ─ | |  |
| Angiolymphatic invasion | Not identified | 163(15) | | 1(ref.) | | ─ | | ─ | | | ─ | | ─ | | ─ | | 163(31) | | 1(ref.) | | ─ | | ─ | | ─ | | ─ | | ─ | |  |
|  | Present | 286(41) | | 1.687  (0.933─3.049) | | 0.083 | | ─ | | | ─ | | ─ | | ─ | | 286(73) | | 1.486  (0.976─2.262) | | 0.065 | | ─ | | ─ | | ─ | | ─ | |  |
| Venous invasion | Not identified | 247(26) | | 1(ref.) | | ─ | | ─ | | | ─ | | ─ | | ─ | | 247(39) | | 1(ref.) | | ─ | | 1(ref.) | | ─ | | 1(ref.) | | ─ | |  |
|  | Present | 202(30) | | 1.515  (0.896─2.564) | | 0.121 | | ─ | | | ─ | | ─ | | ─ | | 202(65) | | 2.296  (1.543─3.417) | | <0.001^***^ | | 1.993  (1.324─3.000) | | 0.001^**^ | | 2.015  (1.334─3.044) | | 0.0009^**^ | |  |
| Perineural invasion | Not identified | 122(10) | | 1(ref.) | | ─ | | ─ | | | ─ | | ─ | | ─ | | 122(19) | | 1(ref.) | | ─ | | ─ | | ─ | | ─ | | ─ | |  |
|  | Present | 327(46) | | 1.717  (0.867─3.404) | | 0.121 | | ─ | | | ─ | | ─ | | ─ | | 327(85) | | 1.758  (1.069─2.891) | | 0.026^*^ | | ─ | | ─ | | ─ | | ─ | |  |
| Combined resection | No | 389(45) | | 1(ref.) | | ─ | | ─ | | | ─ | | ─ | | ─ | | 389(92) | | 1(ref.) | | ─ | | ─ | | ─ | | ─ | | ─ | |  |
|  | Yes | 60(11) | | 1.604  (0.830─3.101) | | 0.160 | | ─ | | | ─ | | ─ | | ─ | | 60(12) | | 0.823  (0.451─1.502) | | 0.525 | | ─ | | ─ | | ─ | | ─ | |  |
| Resection | R0 | 432(51) | | 1(ref.) | | ─ | | ─ | | | ─ | | ─ | | ─ | | 432(98) | | 1(ref.) | | ─ | | ─ | | ─ | | ─ | | ─ | |  |
|  | R1/2 | 17(5) | | 2.764  (1.103─6.929) | | 0.030^*^ | | ─ | | | ─ | | ─ | | ─ | | 17(6) | | 1.846  (0.809─4.210) | | 0.145 | | ─ | | ─ | | ─ | | ─ | |  |
| Proximal margin |  | 447(55) | | 1.002  (0.990─1.015) | | 0.734 | | ─ | | | ─ | | ─ | | ─ | | 447(104) | | 1.002  (0.993─1.012) | | 0.679 | | ─ | | ─ | | ─ | | ─ | |  |
| Distal margin |  | 449(56) | | 1.014  (0.997─1.032) | | 0.096 | | ─ | | | ─ | | ─ | | ─ | | 449(104) | | 1.001  (0.986─1.017) | | 0.864 | | ─ | | ─ | | ─ | | ─ | |  |
| Harvested LN |  | 298(46) | | 0.992  (0.974─1.010) | | 0.386 | | ─ | | | ─ | | ─ | | ─ | | 298(69) | | 0.999  (0.988─1.011) | | 0.899 | | ─ | | ─ | | ─ | | ─ | |  |
| Adjuvant chemotherapy (Missing data, N=120) | No | 59(13) | | 1(ref.) | | ─ | | ─ | | | ─ | | ─ | | ─ | | 59(18) | | 1(ref.) | | ─ | | ─ | | ─ | | ─ | | ─ | |  |
|  | Yes | 270(25) | | 0.384  (0.196─0.751) | | 0.0051^**^ | | ─ | | | ─ | | ─ | | ─ | | 270(58) | | 0.586  (0.345─0.994) | | 0.048^*^ | | ─ | | ─ | | ─ | | ─ | |  |

^*^p<0.05; ^**^p<0.01; ^***^p<0.001.

Abbreviations: ASA, American Society of Anesthesiologists; BMI, body mass index; CEA, carcinoembryonic antigen; CI, confidence interval; DFS, disease-free survival; LN, lymph node; OR, odds ratio; OS, overall survival; ref., reference.
