# Supplementary material for: Favorable short-term oncologic outcomes following laparoscopic surgery for small T4 colon cancer: a multicenter comparative study
Source: World J Surg Oncol. 2020 Nov 13;18:299. doi: 10.1186/s12957-020-02074-5 (PMC7666454; doi:10.1186/s12957-020-02074-5)
Supplement: Supplementary file 2 — Additional file 2. Univariate and multivariate analysis of OS and DFS in patients with tumor size ≤4.0 cm. [file 12957_2020_2074_MOESM2_ESM.docx]

**Additional file 2.** Univariate and multivariate analysis of OS and DFS in patients with tumor size ≤4.0 cm

| Variable |  | OS | | | | | | | DFS | | | | | | |  |
| --- | --- | --- | --- | --- | --- | --- | --- | --- | --- | --- | --- | --- | --- | --- | --- | --- |
|  |  | N(EVENT) | Univariate | | Multivariate (p<0.05) | | Adjusted clinical variable | | N(EVENT) | Univariate | | Multivariate (p<0.05) | | Adjusted clinical variable | | |
|  |  |  | OR (95% CI) | *p*-value | OR (95% CI) | *p*-value | OR (95% CI) | *p*-value |  | OR (95% CI) | *p*-value | OR (95% CI) | *p*-value | OR (95% CI) | *p*-value | |
| Surgery | Open | 29(6) | 1(ref.) | ─ | ─ | ─ | 1(ref.) | ─ | 29(12) | 1(ref.) | ─ | 1(ref.) | ─ | 1(ref.) | ─ | |
|  | Laparoscopic | 88(11) | 0.732  (0.27─1.986) | 0.540 | ─ | ─ | 0.423  (0.145─1.236) | 0.1158 | 88(15) | 0.392  (0.183─0.837) | 0.016^*^ | 0.373  (0.174─0.799) | 0.011^*^ | 0.403  (0.187─0.867) | 0.0201^*^ | |
| Age (years) |  | 117(17) | 1.059  (1.009─1.111) | 0.020^*^ | 1.066  (1.013─1.121) | 0.0143^*^ | 1.077  (1.02─1.137) | 0.0072^**^ | 117(27) | 0.997  (0.966─1.029) | 0.853 | ─ | ─ | ─ | ─ | |
| Gender | Male | 60(9) | 1(ref.) | ─ | ─ | ─ | ─ | ─ | 60(11) | 1(ref.) | ─ | ─ | ─ | ─ | ─ | |
|  | Female | 57(8) | 0.970  (0.374─2.516) | 0.950 | ─ | ─ | ─ | ─ | 57(16) | 1.602  (0.742─3.456) | 0.230 | ─ | ─ | ─ | ─ | |
| BMI (kg/m^2^) | | 117(17) | 0.982  (0.843─1.144) | 0.815 | ─ | ─ | ─ | ─ | 117(27) | 0.885  (0.784─0.999) | 0.048^*^ | ─ | ─ | 0.889  (0.785─1.007) | 0.0632 | |
| ASA score | 1–2 | 115(15) | 1(ref.) | ─ | 1(ref.) | ─ | 1(ref.) | ─ | 115(26) | 1(ref.) | ─ | ─ | ─ | ─ | ─ | |
|  | 3–4 | 2(2) | 22.271  (4.703─105.46) | <0.0001^***^ | 27.871  (5.55─139.967) | <0.0001^***^ | 33.289  (6.047─183.26) | <0.0001^***^ | 2(1) | 13.909  (1.714─112.848) | 0.014^*^ | ─ | ─ | ─ | ─ | |
| Preoperative CEA (10 ng/mL) | | 111(15) | 1.079  (0.751─1.550) | 0.682 | ─ | ─ | ─ | ─ | 111(27) | 1.117  (0.917─1.360) | 0.271 | ─ | ─ | ─ | ─ | |
| Location | Right | 52(9) | 1(ref.) | ─ | ─ | ─ | ─ | ─ | 52(9) | 1(ref.) | ─ | ─ | ─ | ─ | ─ | |
|  | Left | 65(8) | 0.586  (0.226─1.522) | 0.272 | ─ | ─ | ─ | ─ | 65(18) | 1.463  (0.657─3.257) | 0.352 | ─ | ─ | ─ | ─ | |
| Operative time (min) | | 117(17) | 1.004  (0.999─1.009) | 0.110 | ─ | ─ | ─ | ─ | 117(27) | 1.000  (0.994─1.006) | 0.894 | ─ | ─ | ─ | ─ | |
| Blood loss (10 mL) | | 117(17) | 1.006  (0.982─1.030) | 0.646 | ─ | ─ | ─ | ─ | 117(27) | 0.997  (0.97─1.025) | 0.838 | ─ | ─ | ─ | ─ | |
| Transfusion | No | 115(16) | 1(ref.) | ─ | ─ | ─ | ─ | ─ | 115(27) | ─ | ─ | ─ | ─ | ─ | ─ | |
|  | Yes | 2(1) | 4.445  (0.584─33.830) | 0.150 | ─ | ─ | ─ | ─ | 2(0) | ─ | ─ | ─ | ─ | ─ | ─ | |
| Hospital stay (days) | | 117(17) | 1.101  (1.043─1.162) | 0.001^**^ | ─ | ─ | ─ | ─ | 117(27) | 1.078  (1.005─1.156) | 0.035^*^ | 1.081  (1.011─1.155) | 0.022^*^ | 1.089  (1.017─1.165) | 0.0144^*^ | |
| Postoperative morbidity | No | 93(14) | 1(ref.) | ─ | ─ | ─ | ─ | ─ | 93(23) | 1(ref.) | ─ | ─ | ─ | ─ | ─ | |
|  | Yes | 24(3) | 0.864  (0.248─3.010) | 0.819 | ─ | ─ | ─ | ─ | 24(4) | 0.647  (0.224─1.870) | 0.421 | ─ | ─ | ─ | ─ | |
| Conversion | No | 115(16) | 1(ref.) | ─ | ─ | ─ | ─ | ─ | 115(26) | 1(ref.) | ─ | ─ | ─ | ─ | ─ | |
|  | Yes | 2(1) | 4.033  (0.532─30.589) | 0.177 | ─ | ─ | ─ | ─ | 2(1) | 2.555  (0.346─18.862) | 0.358 | ─ | ─ | ─ | ─ | |
| Tumor size (cm) | | 117(17) | 0.658  (0.384─1.129) | 0.129 | ─ | ─ | ─ | ─ | 115(27) | 0.993  (0.611─1.612) | 0.976 | ─ | ─ | ─ | ─ | |
| Nodal status | N0 | 29(2) | 1(ref.) | ─ | ─ | ─ | ─ | ─ | 29(5) | 1(ref.) | ─ | ─ | ─ | ─ | ─ | |
|  | N+ | 88(15) | 2.661  (0.608─11.641) | 0.194 | ─ | ─ | ─ | ─ | 88(22) | 1.650  (0.624─4.359) | 0.313 | ─ | ─ | ─ | ─ | |
| T stage | T4a | 111(16) | 1(ref.) | ─ | ─ | ─ | ─ | ─ | 111(25) | 1(ref.) | ─ | ─ | ─ | ─ | ─ | |
|  | T4b | 6(1) | 1.012  (0.134─7.643) | 0.991 | ─ | ─ | ─ | ─ | 6(2) | 1.466  (0.347─6.198) | 0.603 | ─ | ─ | ─ | ─ | |
| Angiolymphatic invasion | Not identified | 38(3) | 1(ref.) | ─ | ─ | ─ | ─ | ─ | 38(8) | 1(ref.) | ─ | ─ | ─ | ─ | ─ | |
|  | Present | 79(14) | 2.593  (0.743─9.046) | 0.135 | ─ | ─ | ─ | ─ | 79(19) | 1.261  (0.551─2.882) | 0.583 | ─ | ─ | ─ | ─ | |
| Venous invasion | Not identified | 69(10) | 1(ref.) | ─ | ─ | ─ | ─ | ─ | 69(11) | 1(ref.) | ─ | ─ | ─ | ─ | ─ | |
|  | Present | 48(7) | 1.006  (0.382─2.648) | 0.991 | ─ | ─ | ─ | ─ | 48(16) | 2.106  (0.977─4.540) | 0.058 | ─ | ─ | ─ | ─ | |
| Perineural invasion | Not identified | 18(5) | 1(ref.) | ─ | ─ | ─ | ─ | ─ | 18(4) | 1(ref.) | ─ | ─ | ─ | ─ | ─ | |
|  | Present | 99(12) | 0.332  (0.116─0.947) | 0.039^*^ | ─ | ─ | ─ | ─ | 99(23) | 0.783  (0.271─2.265) | 0.652 | ─ | ─ | ─ | ─ | |
| Combined resection | No | 113(15) | 1(ref.) | ─ | ─ | ─ | ─ | ─ | 113(25) | 1(ref.) | ─ | ─ | ─ | ─ | ─ | |
|  | Yes | 4(2) | 3.795  (0.861─16.74) | 0.078 | ─ | ─ | ─ | ─ | 4(2) | 2.835  (0.670─11.990) | 0.157 | ─ | ─ | ─ | ─ | |
| Proximal margin |  | 116(17) | 1.011  (0.970─1.054) | 0.594 | ─ | ─ | ─ | ─ | 116(27) | 1.002  (0.966─1.039) | 0.930 | ─ | ─ | ─ | ─ | |
| Distal margin |  | 117(17) | 1.034  (0.993─1.076) | 0.104 | ─ | ─ | ─ | ─ | 117(27) | 1.006  (0.966─1.047) | 0.789 | ─ | ─ | ─ | ─ | |
| Harvested LN |  | 83(14) | 1.013  (0.972─1.056) | 0.531 | ─ | ─ | ─ | ─ | 83(25) | 0.993  (0.957─1.031) | 0.716 | ─ | ─ | ─ | ─ | |
| Adjuvant chemotherapy (Missing data, N=42) | No | 17(5) | 1(ref.) | ─ | ─ | ─ | ─ | ─ | 17(6) | 1(ref.) | ─ | ─ | ─ | ─ | ─ | |
|  | Yes | 58(6) | 0.218  (0.065─0.733) | 0.0138^*^ | ─ | ─ | ─ | ─ | 58(7) | 0.269  (0.09─0.805) | 0.019^*^ | ─ | ─ | ─ | ─ | |

^*^p<0.05; ^**^p<0.01; ^***^p<0.001.

Abbreviations: ASA, American Society of Anesthesiologists; BMI, body mass index; CEA, carcinoembryonic antigen; CI, confidence interval; DFS, disease-free survival; LN, lymph node; OR, odds ratio; OS, overall survival; ref., reference.
